# Colorectal cancer screening, perceived discrimination, and low-income and trust in doctors: a survey of minority patients

**DOI:** 10.1186/1471-2458-9-363

**Published:** 2009-09-25

**Authors:** Wendi Born, Kimberly Engelman, K Allen Greiner, Shelley B Bhattacharya, Sandra Hall, Qingjiang Hou, Jasjit S Ahluwalia

**Affiliations:** 1 Psychology Department, Baker University, Baldwin City, KS, USA; 2 Department of Preventive Medicine and Public Health, University of Kansas Medical Center, Kansas City, KS, USA; 3 University of Kansas Cancer Center, Kansas City, KS, USA; 4 Department of Family Medicine, University of Kansas Medical Center, Kansas City, KS, USA; 5 Biostatistics Department, University of Kansas Medical Center, Kansas City, KS, USA; 6 Center for Health Equity, University of Minnesota Medical School, Minneapolis, MN, USA

## Abstract

**Background:**

Completion of colorectal cancer (CRC) screening testing is lower among low-income and minority groups than the population as a whole. Given the multiple cancer screening health disparities known to exist within the U.S., this study investigated the relationship between perceived discrimination, trust in most doctors, and completion of Fecal Occult Blood Testing (FOBT) among a low-income, minority primary care population in an urban setting.

**Methods:**

We recruited a convenience sample of adults over age 40 (n = 282) from a federally qualified community health center (70% African American). Participants completed a survey which included measures of trust in most doctors, perceived discrimination, demographics and report of cancer screening.

**Results:**

Participants reported high levels of trust in most doctors, regardless of sex, race, education or income. High trust was associated with low perceived discrimination (p < 0.01). The trend was for older participants to express more trust (p = 0.09) and less perceived discrimination (p < 0.01). Neither trust nor discrimination was associated with race or education. Trust was higher among participants over 50 who were up-to-date on FOBT screening vs. those who were not (31 vs. 29 (median), p < 0.05 by T-test). Among those over 50, up-to-date FOBT screening was nearly associated with high trust (p < 0.06; 95% CI 0.99, 1.28) and low perceived discrimination (p < 0.01; 95% CI 0.76, 0.96). Nevertheless, in multivariate-modeling, age and income explained FOBT completion better than race, trust and discrimination.

**Conclusion:**

Perceived discrimination was related to income, but not race, suggesting that discrimination is not unique to minorities, but common to those in poverty. Since trust in most doctors trended toward being related to age, FOBT screening could be negatively influenced by low trust and perceived discrimination in health care settings. A failure to address these issues in middle-aged, low income individuals could exacerbate future disparities in CRC screening.

## Background

Cancer screening services require some degree of patient compliance with clinical recommendations. With colorectal cancer (CRC) screening, patients voluntarily consent to a specific procedure and complete bowel preparation or home-based testing procedures in order to complete the test. A patient's ability to follow through on these details (i.e., fecal occult blood test (FOBT) screening), may be influenced by the degree to which patients trust medical professionals in general, and physicians specifically, to act in their best interests. Some researchers propose that the level of trust patients place in the medical profession has profound effects on care seeking and completion of preventive, diagnostic, treatment or behavioral recommendations.[1,2]

Trust in the medical profession has received relatively little study in relation to cancer control and screening behaviors amongst the underserved. A general definition of trust typically includes a willingness to place oneself in a position of weakness, exposure, or vulnerability relative to another.[1] Trust in the medical system is a multi-layered process. It involves elements of general system trust, such as faith in medical education and emergency response systems, specific system trust, such as faith in a particular hospital or health plan, general interpersonal trust, such as faith in the goodwill and competence of persons who become health care providers, and specific interpersonal trust, such as faith in the good will and competence of a specific physician.

Researchers investigating trust have, for the most part, focused their efforts on the specific interpersonal trust that individual patients have in the physicians who provide their care. [3-7] Several validated trust surveys have been used in prior research studies such as the 11-item scale adapted from Anderson and Dedrick and validated by Thom et al. asking questions such as "I doubt that my provider really cares about me as a person" and "My provider is usually considerate of my needs and puts them first"[3] Another example is the five-item Wake Forest University Trust Scale used to assess general provider trust issues. The items are: "The doctors at 'this clinic' will do whatever it takes to get patients all the care they need;" "The doctors at 'this clinic' are extremely thorough and careful;" "The medical skills of the doctors here are not as good as they should be;" "You have no worries about putting your life in the hands of the doctors at 'this clinic;" "All in all, you trust the doctors at 'this clinic' completely." A five point scale 1-5 is used, with a higher score indicating a higher level of trust.[7] Some researchers have examined general trust at the level of systems or institutions, including insurance and health care plans,[8] hospitals,[9] and the medical profession in general.[1,10,11] Still other trust researchers have approached the issue from the perspective of patient populations who may perceive themselves to be vulnerable, such as minority patients, [12-18] minority research subjects,[13,19] people who are HIV positive [20] or those at the end of life.[21] If trust does influence health care provision and patient behavior with relation to provider recommendations, it would be more likely to affect preventive care,[22] because illness or pain, unlike most preventive care, might motivate care seeking despite lack of trust.

In a prior study of perceptions of end-of-life care in African American and Latino middle-aged adults, we found what appeared to be a deep lack of confidence in the integrity of physicians and of the health care system in general.[23] Many of the issues raised by participants in this prior study related to conflicts between acting in the best interest of the patient vs. acting to maximize profits. Based on this experience, we incorporated a measure of Trust in Most Doctors (Trust MDs) in a survey we administered to a low-income minority primary care population. Our goal was to evaluate the relationship between Trust MDs and completion of cancer screening. We were also interested in the relationship between reported trust and certain demographic variables such as age, race, income, education and the experience of discrimination.

## Methods

### Study Design

This study was conducted as part of a larger, prospective study assessing completion of colorectal cancer screening in a convenience sample of low-income patients recruited from a large community health center. The survey portion of the study consisted of a 45-minute survey assessing demographics, access to health care, attitudes about health care and specific questions related to cancer and cancer screening.

### Participants

Participants were recruited from the waiting areas of an urban, federally qualified community health center that serves both insured and uninsured patients. Research Assistants approached prospective participants in the waiting areas of the health center, including those for the pharmacy, adult medicine, eye clinic, outreach, information, and the general waiting room of the facility. Participants were adults age 40 years and older who were without acute illness or apparent cognitive deficit.

### Procedures

Research Assistants approached prospective participants in the waiting areas of the health center, asked if they were 40 years of age or older, and then described the study as a 45 minute survey about health care and cancer after which they would receive a $10 retail gift card. Research Assistants then consented and verbally surveyed interested participants in a private room. Full-page cue cards were used for questions that had more than three response options or for questions that benefited from visual cues, such as anchored Likert scales.

The survey instrument included 129 items and took approximately 45 minutes to complete. The first 99 survey items assessed demographic information, access to healthcare, perceived discrimination, cancer fatalism, trust in health care providers, diet, physical activity, smoking, breast or prostate cancer screening, and knowledge of colon cancer screening tests and guidelines. Additional items assessed prior receipt of, preferences for, and barriers to FOBT and endoscopic screening. The Human Subjects Committee from the University of Kansas Medical Center approved the study prior to commencement of the project.

### Analyses

We reviewed literature relevant to trust in the medical system to find items conceptually related to focus group data.[23,24] Our survey questions were modified from the identified existing measures. Questions were designed to measure trust in the areas of physician honesty, commitment to patient welfare, professional competence, confidentiality of medical information, and research integrity to determine the relationship between self-report of trust and self-report of important preventive health behaviors, such as cancer screening. We modified items to target opinions about "most physicians". The resulting scale of Trust in Most Doctors (Trust in MDs) included eight items (alpha = 0.85). One item was written by the research team, the others were adapted from items developed by Kao et. al. (5 items),[5,25] Corbie-Smith et. al. (1 item),[26] and Safran et. al. (1 item),[6] Items were chosen to assess trust in most physicians' ability to provide the following elements of quality care: *commitment *to the welfare of the patient (2 items; e.g., "Most doctors will try to help someone who is sick, even if the person has no way to pay for the care".), the technical *competence *to protect that welfare (2 items; e.g., "Most doctors can be trusted to refer patients to a specialist when needed".), *honesty *(1 item; "Most doctors can be trusted to give patients information on all medical options and not just options that are covered by the health plan".), respect for patient *confidentiality *(1 item; "Most doctors can be trusted to keep personally sensitive information private".), *research *integrity (1 item; "Most doctors would not ask a patient to participate in medical research if they thought it might harm the patient".), and *global *quality of care (1 item; "Most doctors can be trusted to offer high-quality medical care".). Responses to these items were given on a four-point Likert scale (Agree; Somewhat Agree; Somewhat Disagree; Disagree). Responses for items were summed to create a composite score reflecting trust in most doctors. Trust scores could range from 4 (low trust) to 32 (high trust).

The experience of discrimination was assessed using a brief, four-item version of David Williams' Perceived Discrimination scale. The full, nine-item scale has been extensively validated. [27-29] Because the distributions of Trust in MDs and Perceived Discrimination were highly skewed, we split scores at the median and compared those who were relatively low on each variable to those who were relatively high.

The survey included ten demographic questions taken from the 2001 Behavioral Risk Factor Surveillance System (BRFSS) survey which assessed age, sex, race, income, education, and employment;.[30] nine questions taken from the 2002 Medical Expenditures Panel Survey.[31] and the 2002 BRFSS.[32] which addressed general health and access to care questions; and a series of questions about cancer screening. Up-to-date screening for colon cancer was defined as the report of FOBT within the last two years.

### Data Management

Surveys were double entered and exported from Microsoft Access into SAS version 8. When data were reconciled, range and logistic checks were performed to ensure the accuracy of the database.

### Data Analysis

We calculated means and frequencies for all study variables. We used Spearman rank-order correlation to determine how demographic, attitudinal, and preventive behavior variables related to each other and to make decisions about further modeling. We conducted multivariate analysis (including all variables correlated at a p < 0.20) to determine the items associated with self-report of FOBT completion among the sample.

## Results

Participants included 293 adults 40 years of age or older, equally divided between males and females (Table 1). Of our sample, 53% were age 40-49 with the remaining 47% aged 50 or older. The majority of participants (69%) reported being African American, 71% had incomes less than $1200 per month, and 44% reported being without insurance or Medicaid coverage. Of the 300 surveys initiated, seven were excluded: three were incomplete; two were duplicates; and two were not members of the target population. Due to missing values, 282 participants with complete data were used for analyses.

**Table 1:** Participant Demographics (N = 282)

	**Category**	**Age < 50** **(N = 149)** **n (%)**	**Age ≥50** **(N = 133)** **n(%)**
Gender	Female	74 (49.6%)	72 (54.1%)
Race/Ethnicity	African American	111 (74.5%)	85 (63.9%)
	Hispanic	3 (2.1%)	3 (2.3%)
	Asian	0 (0%)	2 (1.5%)
	Native Hawaiian of Pacific Islander	1 (0.7%)	0 (0%)
	American Indian or Alaska native	3 (2.1%)	2 (1.5%)
	White	26 (17.5%)	36 (27.1%)
	Other	4 (2.7%)	2 (1.5%)
	Refused or Don't Know	1 (0.7%)	3 (2.3%)
Annual Income	<40K	110 (73.8%)	95 (71.4%)
	> = 40K	39 (26.2%)	38 (28.6%)
Insurance	Yes	80 (51.9%)	82 (60.3%)
Marital Status	Married	19 (13.0%)	24 (18.1%)
	Divorced	38 (25.5%)	56 (42.1%)
	Widowed	9 (6.0%)	13 (9.8%)
	Separated	24 (16.1%)	17 (12.8%)
	Never Married	44 (29.5%)	18 (13.5%)
	Living with significant other or partner	15 (10.1%)	5 (3.8%)
Education	Grades (0-11)	38 (25.5%)	41 (30.8%)
	Less Than High School	67 (45.0%)	37 (27.8%)
	GED or High School	28 (18.8%)	30 (22.6%)
	College 1~3 years	9 (6.0%)	13 (9.8%)
	College Graduate (4-year degree)	4 (2.7%)	9 (6.8%)
	Graduate Degree	3 (2.0%)	3 (2.3%)

**Figure 1:**
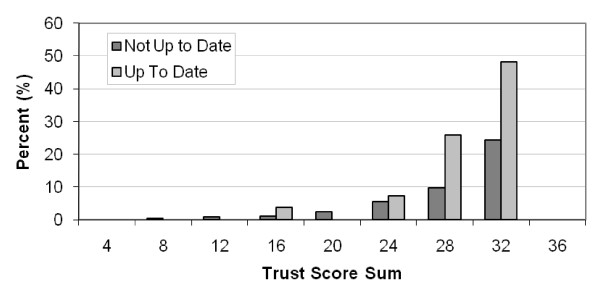
**Trust Scores and Fecal Occult Blood Testing Status among Participants ≥50 Years of Age**.

Among participants who were 50 years of age or older (n = 136), trust in MDs was generally high (mean = 27.60; median = 29, STD = 5.14, range = 8, 32). Trust in MDs was higher for those completing FOBT (n = 23, mean = 29.47; median = 31, STD = 3.95, range = 16, 32) compared to those who did not complete FOBT (n = 113, mean = 27.22; median = 29, STD = 5.29, range = 8, 32) (Figure 1.)

Of the 282 participants, trust in MDs also was generally high (mean = 26.96; median = 29, STD = 5.28, range = 8, 32), resulting in a ceiling effect for trust scores. As in the 50 year old and older subsample, trust in MDs was higher for those who completed FOBT (n = 27, mean = 28.56; median = 31, STD = 5.27, range = 14, 32) compared to those who did not complete FOBT (n = 255, mean = 26.79, median = 29, STD = 5.27, range = 8, 32). Perceived discrimination was similarly distributed, with most people reporting low levels of discrimination (mean = 7.27; median = 7, STD = 5.61, range = 0, 20).

Trust in MDs was negatively associated with perceived discrimination (r = -.25, p < 0.01) and trended toward being positively associated with age (r = .10, p = 0.09). Perceived discrimination was negatively associated with both age (r = -.24, p < 0.01) and income (r = -.21, p < 0.01). For participants aged 50 and older, neither trust nor discrimination was associated with race (African American vs. Caucasian, 29 vs.28 (median) with p = 0.9 and 6 vs.7 (median) with p = 0.2 by T-test) or years of education (p = 0.09 and 0.47 by spearman correlation analysis). Participants 50 years of age or older who were up-to-date on FOBT screening trended toward being more trusting of physicians (Figure 1). In a logistic regression with participants aged 50 and older, up-to-date FOBT screening was not quite associated with higher trust (OR = 1.13, p = 0.06, 95% confidence interval 0.99, 1.28), but was associated with lower perceived discrimination (OR = 0.86, p < 0.01, 95% confidence interval 0.76, 0.96). Despite the reduced range, age remained associated with both trust (r = .39, n = 133, p < 0.01) and perceived discrimination (r = -.30, n = 133, p < 0.01).

Multivariable logistic regression showed age and income to be related to reported FOBT adherence among those age 50 years old or greater (Table 2).

**Table 2:** Multivariate logistic regression for effect on probability of up-to-date FOBT among Participants ≥50 Years of Age

		**Standard**		**Wald**	
		
**Parameter**	**DF**	**Estimate**	**Error**	**Chi-Square**	**Pr > ChiSq**
Intercept	1	-7.8199	2.0945	13.9395	0.0002
MDs	1	0.0617	0.0669	0.8520	0.3560
Age	1	0.0631	0.0288	4.7938	0.0286
Income	1	0.0237	0.0137	2.9822	0.0842

## Discussion and Conclusion

In this study, trust in most doctors reliably co-varied with perceived discrimination but not with race/ethnicity. This suggests that lack of trust in doctors was associated with the experience of being treated unfairly or inconsiderately, but not linked perceptually with race/ethnicity. The relationship between income and perceived discrimination found in this study is important because of the drastically reduced range of incomes represented in this sample. The vast majority of participants fell below federally designated poverty levels. All study findings that relate to income in this sample are likely to be more robust and easily detected in samples with a wider range of incomes.

The results of this study suggest the relationship between trust and reduced completion of preventive screening is not direct. The relationships between trust, perceived discrimination and adherence to different preventive behaviors may be quite complex, and may be influenced by variables such as income and access to care more than race. Although there is debate about whether or not trust in our system of health care can best be improved by encouraging change at the patient, provider, system, societal, social policy, or governmental level, [33-36] there is consensus that improving trust would benefit the health of the public, especially those populations that have particularly low trust in health care. Trust in MDs may play a completely different role in preventive adherence in populations for whom perceived discrimination is minimal. Further longitudinal investigation could explore more directly the likely link between trust in MDs with willingness to follow physician recommendations for preventive screening and demographic variables. Longitudinal studies could also address the relationship between trust, discrimination, and age to determine whether Trust in MDs is a function of an age cohort (or of historical events), or whether it changes gradually over time as people have increasing experiences with the medical system and health problems.

This study had several limitations. The sample size was small, over age 40, and drawn from a single source, resulting in a restricted range of participant ethnicity, willingness to seek care, and age. Sample selection bias, being from a busy outpatient clinic, likely contributed to the high level of trust observed, and the skewed distribution of participant income. In addition, although our survey included questions from validated surveys, our specific trust scale was not evaluated in terms of its measurement properties. Future studies should draw participants randomly, from all income levels, and racial/ethnic backgrounds, with no association to provision of health care. Doing so would allow for meaningful comparisons of trust in MDs, perceived discrimination, and FOBT completion. In a larger, more diverse sample, effects of age, income, and education will be more easily detected, and relationship of these socio-demographic variables to trust and discrimination could be explored. A study of how trust develops over time, beginning in early adulthood, and the relationship between trust and adherence to preventive care would also help guide future preventive interventions.

Colorectal cancer screening may be fundamentally different from other types of cancer screening, with differentiating barriers and facilitators.[24,37] CRC screening recommendations from physicians are relatively unlikely to be adhered to, especially among the underserved.[38] Unlike many screenings, FOBT requires more patient cooperation than just showing up for, or even preparing for an appointment that has already been set. It is common for clinicians to simply order, schedule, or perform mammograms or pap smears. In contrast, FOBT kits must be completed by the patient, yet are often handed out with only written instructions, little discussion and no follow-up for non-return. Lieberman et al. described the need of a FOBT "program" to maximize its success. The program consisted of proper performance of the test, adherence and follow-up of positive and negative results. Ultimately, the last element of this program was to provide proper cancer care for detected cancers.[39] Thus, FOBT requires significant, unprompted behavioral initiation and follow-through on the part of the patient. Moreover, there is little to reinforce FOBT completion: there is no person-to-person interaction, no medical technology, no prompts, and often, no feedback if results are negative. This system creates a number of opportunities for the necessary patient behavior to be disrupted, diminishes the perceptual importance of the results, and gives the impression that the health care providers are not especially concerned with adherence.

These difficulties for FOBT screening leave the door open for trust in physicians and perceived discrimination to play a larger role in CRC screening completion relative to other types of screening. Affordability and ease of use make FOBT screening the most readily available form of CRC screening among the underserved. This study provides initial evidence to suggest that perceived discrimination and trust are related to FOBT completion, although all three may be a function of poverty. Despite the role of poverty, it is possible that the decreased perceptions of discrimination, or increased trust in physicians could counteract some of the negative correlates of reduced access to care. Because the constructs of trust and perceived discrimination overlap both theoretically and statistically, it is possible that efforts to address one, could have effects on the other. Interventions to increase FOBT screening rates and reduce disparities in CRC mortality among the underserved would do well to consider factors that likely contribute to both trust in doctors and the perception of discrimination, such as income, rather than focus narrowly on race.

## Financial Competing interests

The authors declare that they have no competing interests.

## Authors' contributions

KAG, JA, and WB designed the study. WB, KE, KAG, & SB significantly contributed to the manuscript writing. SH and QH completed all statistical analyses.

## Pre-publication history

The pre-publication history for this paper can be accessed here:


